# Influence of Film Role on the Positive Development of College Students’ Entrepreneurial Behavior Using Positive Psychology

**DOI:** 10.3389/fpsyg.2022.843708

**Published:** 2022-06-30

**Authors:** Hang Zhang, Huizhen Long, Yinchang Chen

**Affiliations:** ^1^School of Media and Design, Hangzhou Dianzi University, Hangzhou, China; ^2^School of Hotel and Tourism Management, The Hong Kong Polytechnic University, Hong Kong, Hong Kong SAR, China; ^3^School of Media and Law, NingboTech University, Ningbo, China

**Keywords:** multiculturalism, positive psychology, inspiration movies, college student entrepreneurship, education in higher institutions

## Abstract

To help College Students improve their entrepreneurial psychology and enrich their innovative achievements, this paper investigates College Students’ entrepreneurial behavior based on the theory of positive psychology under the background of multiculturalism. Firstly, the background and development status of multiculturalism are expounded, and the main features of the multicultural era are pointed out; Secondly, the current situation is explained for College Students’ mental health and entrepreneurial behavior, and the specific connotation of College Students’ entrepreneurial psychology is summarized according to entrepreneurial behavior, including entrepreneurial consciousness, entrepreneurial will, entrepreneurial capability, and entrepreneurial personality; Thirdly, the positive effects of inspirational films and roles on the psychology of College Students are discussed. From the connotation of film and television culture, the main features of films are reviewed: intuitiveness, immediacy, universality, entertainment, and orientation. Finally, according to the psychological impact of inspirational films on College Students, a Questionnaire Survey (QS) is designed and distributed among the local College Students, and assumptions are put forward. The results show that the differences of College Students’ entrepreneurial level are mainly reflected in gender, urban, and rural household registration, only-child-or-no, and entrepreneurial experience, and the gender differences are mainly in entrepreneurial consciousness, will, and personality (*p* < 0.05); There are significant differences in consciousness, will, capability, and personality between urban and rural household registration and entrepreneurial experience (*p* < 0.05); The differences between the only-child and non-only-child are obvious in three aspects: will, capability, and personality (*p* < 0.05). After the intervention of inspirational films, the average scores of all dimensions of the entrepreneurial level of students in the intervention group are 35.45, 36.41, 38.17, and 37.22, respectively, which have a certain improvement compared with the situation before the intervention. There are significant differences in the four dimensions (*p* < 0.05), which is statistically significant, indicating that the intervention of inspirational films has a positive impact on students’ entrepreneurial level.

## Introduction

China’s Reform and Opening-up have witnessed a social transformation, in which the original social interest pattern has been broken, people’s interest consciousness has been awakened, and the vitality of the whole society has been stimulated; Further, the cultural pattern of China’s society has also undergone a fundamental change, and the original unified cultural structure has begun to loosen (Helen et al., 2021). In this context, people’s ideas have changed profoundly. College Students, as the most receptive and active group, are deeply influenced by multiculturalism (Deng et al., 2021). The cultivation and maintenance of College Students’ political identity are facing new challenges. College Students’ successful entrepreneurship relies on national preferential policies and social support, as well as an excellent psychological quality (Jardim, 2021), especially entrepreneurial Psychological Capital (PsyCap). In recent years, Innovation and Entrepreneurship Education (IEE) for College Students in China is more about introducing new entrepreneurial fields, providing more entrepreneurial opportunities (Liu et al., 2019), building a broader entrepreneurial platform, and teaching knowledge about entrepreneurial projects (Perera et al., 2022). According to the research, the diversified cultural information environment created by the government can greatly stimulate the entrepreneurial passion of entrepreneurs and promote successful entrepreneurship (Feng and Chen, 2020). Currently, the cultivation of College Students’ entrepreneurial PsyCap has been rarely involved, without which IEE lacks comprehensiveness. In the process of entrepreneurship, due to the lack of education on entrepreneurial PsyCap (Carpenter and Wilson, 2021), College Students are prone to psychological fluctuations, resulting in College Students losing confidence in entrepreneurship, and some even choose to give up (Wu et al., 2020). Therefore, it is necessary to strengthen the cultivation of College Students’ entrepreneurial PsyCap and enable College Students to form positive entrepreneurial PsyCap. Studies show that Psychological Health (PsyH) education plays a crucial role in helping College Students’ entrepreneurial PsyCap and can provide guarantee and support for entrepreneurship in the future (Liu et al., 2021).

In the period of information explosion in modern society, media are all-around people’s life and work, and teenagers belong to a special group in the media environment. Due to their physio-psychological peculiarities and their special needs for the media environment (Lavelle, 2021), teenagers are strongly affected by media information. For example, media information helps teenagers shape cognitive systems and develop positive personalities (Tiernan and Deveci, 2021). Therefore, as a new educational force, the media have become an important way to affect the positive growth and development of teenagers (Zelin et al., 2021). In the information age, the influence of the developing media has been paid ever-more attention by the public. Under the influence of various forms of media, the influence of film is more direct and profound (Lans et al., 2021). The development of film will have a certain impact on people’s way of thinking, behavior, and lifestyle (Vidmar, 2020).

Thus, innovatively, the inspirational films intervention is studied under an independent sample analysis of College Students’ entrepreneurial psychology. The QS method is combined to investigate and collect evidence. The proposal has a vast development space in the current field and has an essential reference and guiding significance for the college IEE.

### Recent Work

#### College Students’ Entrepreneurial Psychological Capital

Since 1920, British scholars have begun to study entrepreneurial quality and entrepreneurial psychology. After over a century of development, their theoretical achievements and practical education experience have matured (Ayob, 2021). Alfred Marshall is the first to define the concept of entrepreneurial quality globally. He believes that entrepreneurial quality is the psychological desire to create things with bold and keen psychological characteristics, enterprising, and a strong desire for success (Carpenter and Wilson, 2021). Joseph Peter believes that the quality of entrepreneurship and innovation is the sum of all the elements needed by the entrepreneurial subject. The analysis results in “investigating the factosrs affecting the entrepreneurial psychology of graduate students in Tehran University” show that physiological factors, education level, university, and age explain 63.5% of the variance of dependent variables, respectively. At the same time, the test results suggest that there are significant differences in the independent variables of marital status, invention, occupation, father’s education level, organization, and entrepreneurial mentality (Daniella et al., 2021).

The research results of foreign countries on cultivating entrepreneurial quality and entrepreneurial PsyCap have important enlightenment and reference significance for domestic research on College Students’ entrepreneurial PsyCap. However, its beneficial experience, theories, and methods must be used in principle and pertinence according to China’s basic national conditions and the characteristics of College education (Li et al., 2021).

At present, domestic scholars have made many studies on College Students’ entrepreneurial psychology. Wu et al. (2020) believed that entrepreneurial psychology was closely related to personal consciousness. Strengthening personal consciousness through deliberate training would help improve the success rate of entrepreneurship. For example, people could enhance their entrepreneurial PsyCap through subconscious training, self-suggestion, and local success, conducive to entrepreneurs forming good entrepreneurial PsyCap and stimulating entrepreneurial motivation in competition (Wu et al., 2020). Chen (2019) regarded cultivating entrepreneurial PsyCap as one of the important contents of IEE, which mainly includes independence, boldness, tenacity, cooperation, and meticulousness (Ji et al., 2020). Out of them, IEE in American higher institutions represented by Baisen business school and Harvard University focused on incorporating entrepreneurship into the concept of IEE and encouraged innovation and entrepreneurship by building a positive campus culture. They established a policy-sound IEE practice system, encouraged students to participate in practical experience activities, and strengthened the guidance and education of the industry, university, research, and application, as well as the training of entrepreneurs’ psychological quality (Chen, 2019). Liu and Chen (2021) claimed that cultivating College Students’ entrepreneurial PsyCap could increase College Students’ entrepreneurial success rate (Wu et al., 2020). By analyzing the current situation and causes of College Students’ entrepreneurial PsyCap, they put forward the following targeted ways. (1) Setting up entrepreneurial PsyCap training courses and implementing comprehensive guidance. (2) Combination of entrepreneurship guidance and psychological guidance. (3) Improve self-education ability and enhance interpersonal skills training. (4) Improve students’ psychological guidance and enhance students’ adaptability. (5) Strengthen teaching practice and cultivate strong will (Liu and Chen, 2021).

#### Influence of Movies on College Students’ PsycH

The theory of developmental psychology holds that College Students are in the early stage of adulthood. During this stage, they absorb knowledge and experience in many aspects and develop self-consciousness rapidly. Due to the physical and psychological maturity at this stage, coupled with the expectations and pressures from all social aspects and cultural factors, individual self-concept takes shape into a complex multi-dimensional and multi-level psychological structure (Patel, 2021). Self-evaluation is an individual’s manifestation of a more independent, unique, and stable psychological character. Self-experience also contains rich content, and the inner experience related to self is more profound and sensitive. At this stage, individual values and outlook on life begin to form, which are affected by many factors in socialization. Psychologist Milton Rockucci emphasizes that various social factors, including social culture, school, and family, have an impact on the formation of College Students’ values.

In view of film’s impact on College Students’ PsycH, the current research mostly focuses on film education and film therapy. In the research field of film education, Li et al. (2020) compared four training methods: film course, group training, a psychology course, and e-learning. They confirmed that the effect of film courses on College Students’ peer trust training was significantly better than the latter, which was the best way of interpersonal training (Li et al., 2020). Hasan and Bao (2020) proved through experiments that the use of film text in psychological education could significantly promote students’ PsycH, psychological maturity, psychological stability, and social adaptability. The film provided a feasible solution for College Students’ PsyH education (Hasan and Bao, 2020).

Here, in the context of multiculturalism, the entrepreneurial psychology of College Students is studied using positive psychology. So far, most studies in culture and mental health only combine film appreciation with theoretical courses rather than qualitatively study the influence mechanism (O’hUigin et al., 2021).

## College Students’ Entrepreneurial Behavior Based on Positive Psychology

### Multicultural Background

The feature of multiculturalism includes many dimensions. First, diversity: diversity is the primary feature of multiculturalism, which is manifested through the prefix “multi,” meaning diversity; that is, multiculturalism is the coexistence of multiple cultures. Second, inclusiveness (Agboola et al., 2021) is the second most significant feature of multiculturalism. The coexistence of multiple cultures is the result of the inclusiveness of multiculturalism. Although multiple cultures are individually distinct, they are not contradictory. Multiculturalism is the mutual tolerance of various cultural forms. Third, integration: multiculturalism is diverse and inclusive, which also shows that the integration of multiculturalism is the basic law of cultural development. In inclusiveness, multi civilization shows that one culture and other cultures absorb the essence and influence each other, and integrate into communication and collision (Yvonne, 2020).

### PsycH of College Students

College Students are the advanced elements and elite group among China’s youth, “a very valuable talent reserve for the country and society,” and the mass youth foundation of the Communist Party of China (CPC)’s long-term governance. Only with the healthy growth of College Students can the Chinese nation have hope, and socialist undertakings have a future. College Students are talents trained by socialist universities, undertake special historical missions, and are related to the future development of socialism with Chinese features (Maha et al., 2021). College Students are not only the recipients of a country’s mainstream culture, especially the mainstream political culture, but also the disseminators of Chinese mainstream culture. The status of College Students’ political identity reflects the basic situation of a country’s higher education serving politics, as well as the stability of national political power. The status of College Students’ political identity represents the future trend of social and political identity and occupies an extremely important position in the political identity system of the whole society (Xing, 2021). Psychological quality refers to stable psychological features formed at a person’s spiritual level through the interaction of cognition, emotion, will, and personality and plays a guiding role in people’s psychological and practical activities (Adarsha et al., 2021).

### College Students’ Entrepreneurial Psychology

**Figure 1:**
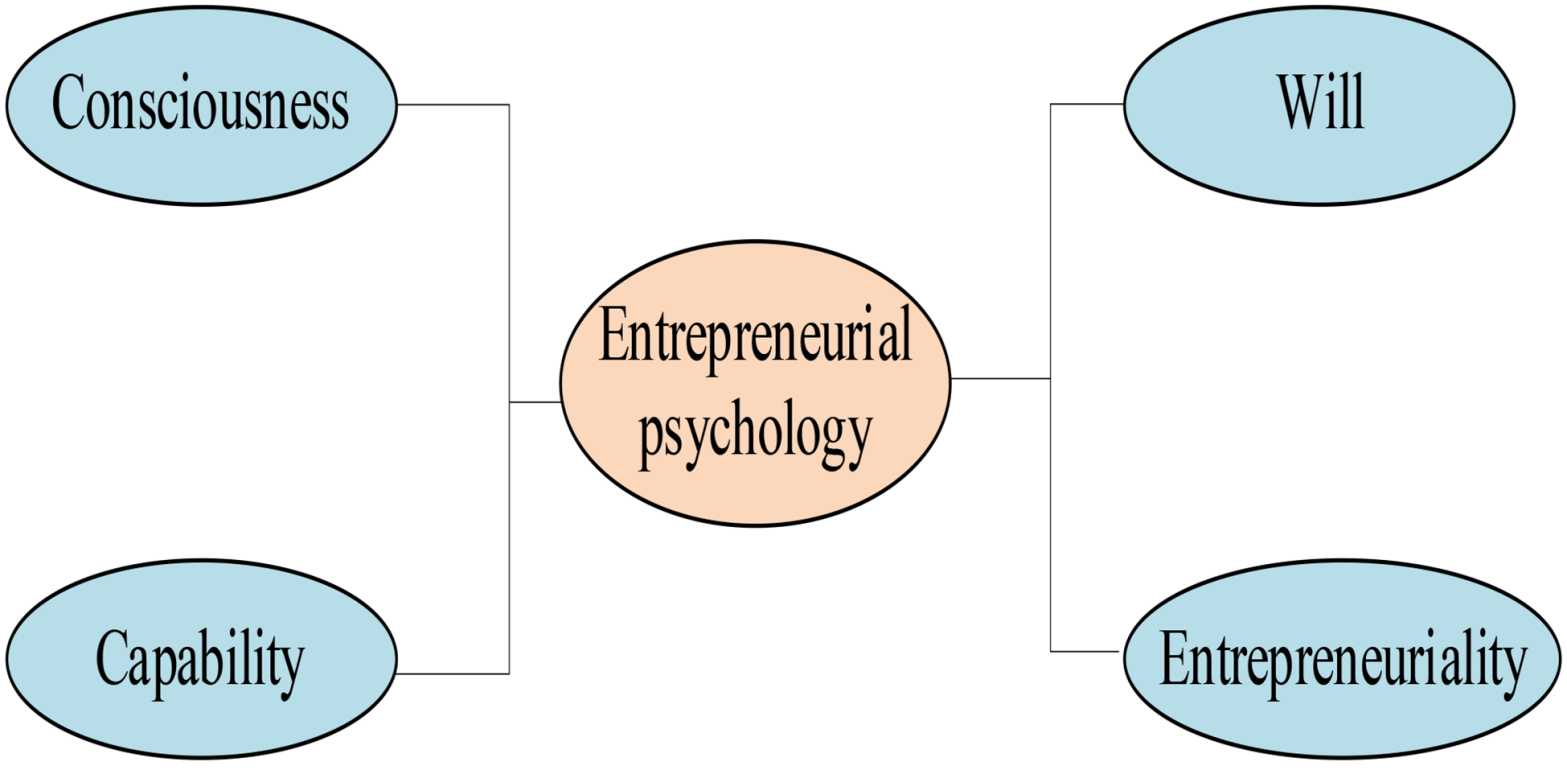
Four dimensions of entrepreneurial psychology.

College Students’ entrepreneurial PsyCap is a stable personality psychological feature gradually formed by the interaction between College Students and their own actions under the influence of environment and education in the process of growth and will be continuously displayed in entrepreneurial activities (Volkova, 2021). Under the guidance of positive psychology, College Students have been successfully cultivated with positive entrepreneurial PsyCap by giving full play to the leading power of higher institutions and integrating various effective forces, such as social, family, and personal strength (Lu et al., 2021). It mainly cultivates College Students with positive entrepreneurial awareness, good entrepreneurial capability, tenacious, entrepreneurial will, and unique entrepreneurial personality, as shown in Figure 1.

“Entrepreneurial consciousness is a kind of expectation of entrepreneurial subjects and a personal psychological tendency to adhere to entrepreneurial activities, including psychological components, such as needs, motivation, interest, thought, belief, and world outlook (Hongbin and Juan, 2021).”

Will is a unique psychological phenomenon of human beings. It is a stable and continuous psychological state formed by a person to achieve his goal. Entrepreneurial will means that the entrepreneurial subject decides to achieve the entrepreneurial goal, strive to control and adjust their mentality, independently solve the difficulties on the entrepreneurial road, and form a continuous and stable psychological state to realize the entrepreneurial dream (Mir and Alarifi, 2021). Entrepreneurial will is generally manifested as tenacious struggle, willingness to forge ahead, self-reliance, and perseverance (Yuan and Wu, 2020).

Entrepreneurial capability refers to the ability of entrepreneurs to develop or create a new research field or creative things by integrating their knowledge, experience, and skills, and show a continuous and stable psychological process during entrepreneurship (Naveed and Zeinab, 2021).

Entrepreneurial personality is a stable psychological quality formed based on physiological quality and under the influence of education and social practice. Entrepreneurs with entrepreneurial personalities will always move forward bravely, not afraid of difficulties and dangers, and feel the value and significance of their existence in the process of entrepreneurship.

### The Positive Influence of Inspirational Films and Roles on College Students

With the deepening of positive psychology in school PsycH education, it is urgent to develop positive teenagers from multiple angles. With the impact of multicultural information and the development of media, film, and television works play an increasingly important role in daily education (Martínez et al., 2021). According to previous studies, watching movies accounts for a high proportion of teenagers’ after-school life. The excellent qualities embodied in the scenes and characters in the film often deeply infect the viewers, and these excellent fragments can also become a new model of school psychological education (Murray, 2017). Investigation and research suggest that the dissemination of films has a very significant impact on teenagers’ thoughts and behaviors, value orientation, interests, and hobbies, which are worthy of attention and thinking (Weicht and Jónsdóttir, 2021).

During teenagers’ growth, their way of thinking and choice of values are closely related to their social environment. In today’s open information society, the coverage and influence of films on teenagers are gradually expanding and are becoming one of the important ways to affect teenagers’ positive growth and development. From the meaning of film and television culture, their features are summarized below.

#### Intuitiveness

Film culture interprets the life and reflects hot spots with its realistic and vivid image reproduction so that viewers can get a true understanding and feel from intuitive audio-visual. The film is straightforward and vivid and uses image and sound to directly act on people’s senses: vision and hearing, thereby conforming to the habit of human beings to feel objective things (Zheng and Liu, 2020).

#### Immediacy

It is mainly the interpretation of film characters and plots to chase various social hot spots and humanistic forms, thereby keenly capturing and reproducing the theme. It can meet the psychological needs of viewers to absorb the latest social trends to the greatest extent.

#### Universality

It refers to the universality of the scope of film communication, which is limited to the immediacy of the film. At the same time, the expression form and content of the film should not be too complex but should be concise, clear, and popular. This will expand the spread and acceptance of the film.

#### Entertainment: Film Culture Also Has Strong Entertainment

The film is different from boring words. Although it does not have the strict logic and profound connotation unique to words, it has a vivid image that words cannot show.

#### Orientation

The results of film dissemination show that film culture is also significantly demonstrative and oriented. Due to the socialization of movie content, its exemplary nature to the viewer’s sociality has largely replaced traditional education (Bamufleh et al., 2021).

Research results indicate the inspirational film *The Pursuit of Happiness* has the most noticeable impact on College Students’ PsyH and coping style regarding problem-solving, help-seeking, and retreat. After watching movies, College Students prefer to adopt mature coping styles, such as problem-solving and help-seeking against emergencies and harsh conditions. By comparison, they are less likely to choose immature coping styles, such as retreat. Probably, the characters’ experience in the film has had a positive impact on College Students. With the theme of pursuing dreams and taking responsibility, the course can promote the change of College Students’ coping style through film appreciation and subsequent discussion and sharing.

After enjoying the film, *The Shawshank Redemption*, College Students’ self-confidence against difficulties and environmental challenges has been significantly enhanced. Presumably, the film narrates a rich plot to show the strong spiritual power of the protagonist Andy’s self-redemption, which also appeals to the College Students watching the film. The theme of the course is hope and spiritual salvation. After the film appreciation process, students are guided to discuss and share. It can also be found that College Students’ sense of self-efficacy has strengthened significantly.

### Questionnaire Design

The film is a comprehensive art with rich artistic forms and strong emotional appeal. Some classic film roles can bring great influence to film viewers. As the main force of the young generation, college students live in an era of new media prosperity, when numerous excellent film works are emerging continuously. Watching inspirational films can broaden their horizons and establish a positive outlook on life, values, and world outlook. At the same time, good personal cultivation can be shaped, and perseverance can be cultivated to improve personal entrepreneurial PsyCap and improve the success rate of entrepreneurship. This is a new way for film works as College Students’ IEE. Therefore, from the perspective of positive psychology, inspirational films are selected to study College Students’ entrepreneurial psychology and discuss the influence of film roles on College Students from four aspects of entrepreneurial psychology. The assumptions proposed are listed in Table 1.

**Table 1:** Research assumption.

	Research assumptions
1	Inspirational film roles have a significant impact on College Students’ entrepreneurial PsyCap.
2	It is necessary and feasible to supplement the content of inspirational film appreciation in the course of PsyH education.

#### Research Methods

To study the impact of inspirational films on College Students’ entrepreneurial psychology, this paper will use QS and intervention methods to investigate College Students. The content refers to the QS of College Students’ Entrepreneurial PsyCap Scale. Additionally, during the investigation, the research objects are divided into the intervention group and control group through random selection, thereby ensuring that the psychological quality level of the two groups of students is equal and balanced. Meanwhile, the number and age of different genders should be equally distributed among the two groups. Before the experiment, the two groups of students are pre-tested on their entrepreneurial psychological level, and then the intervention experiment of entrepreneurial, inspirational films is carried out on the intervention group. The students in the intervention group are allowed to watch inspirational films, such as *Qian Xuesen*, *Chinese Partners*, and *Forrest Gump*, while the control group does not watch films. After the intervention, the entrepreneurial psychological level of the two groups of students is measured.

#### Research Object

This QS is distributed for College Students in local University C. Two classes are randomly selected in the same grade for QS. Each class contains 30 students, with ages between 18 and 20. Class A is the intervention group, and class B is the control group. In the experiment, eight class hours of inspirational film appreciation course will be added to class A, while the students in the control group will not have a film appreciation course. At the end of the semester, QS is conducted to analyze the impact of inspirational films on the students in the experimental group. Additionally, in the experiment, on top of the short-term longitudinal research to explore the impact of inspirational film courses on students’ PsycH level, the specific psychological changes of students are understood through interviews, and finally, all the QS data are sorted out together with the interview materials. The results of the QS are evaluated through the five-point scoring method, in which five points, four points, three points, two points, and one point, respectively, represent the five degrees of very compliance, compliance, general, non-compliance, and very non-compliance. The higher the score is, the higher the level of entrepreneurial psychology is.

#### Reliability of QS

Usually, people are used to using the internal consistency (Cronbach *α*) coefficient to evaluate the reliability of the QS, and (*α* coefficient > 0.7) indicates that the QS has good reliability. After the QS data are input into SPSS22.0 for analysis, the *α* coefficients of different dimensions can be obtained, which are greater than 0.7, indicating that the QS has high reliability.

## Analysis of Research Results

### Analysis of Pre-test Results of Entrepreneurial Psychology

**Figure 2:**
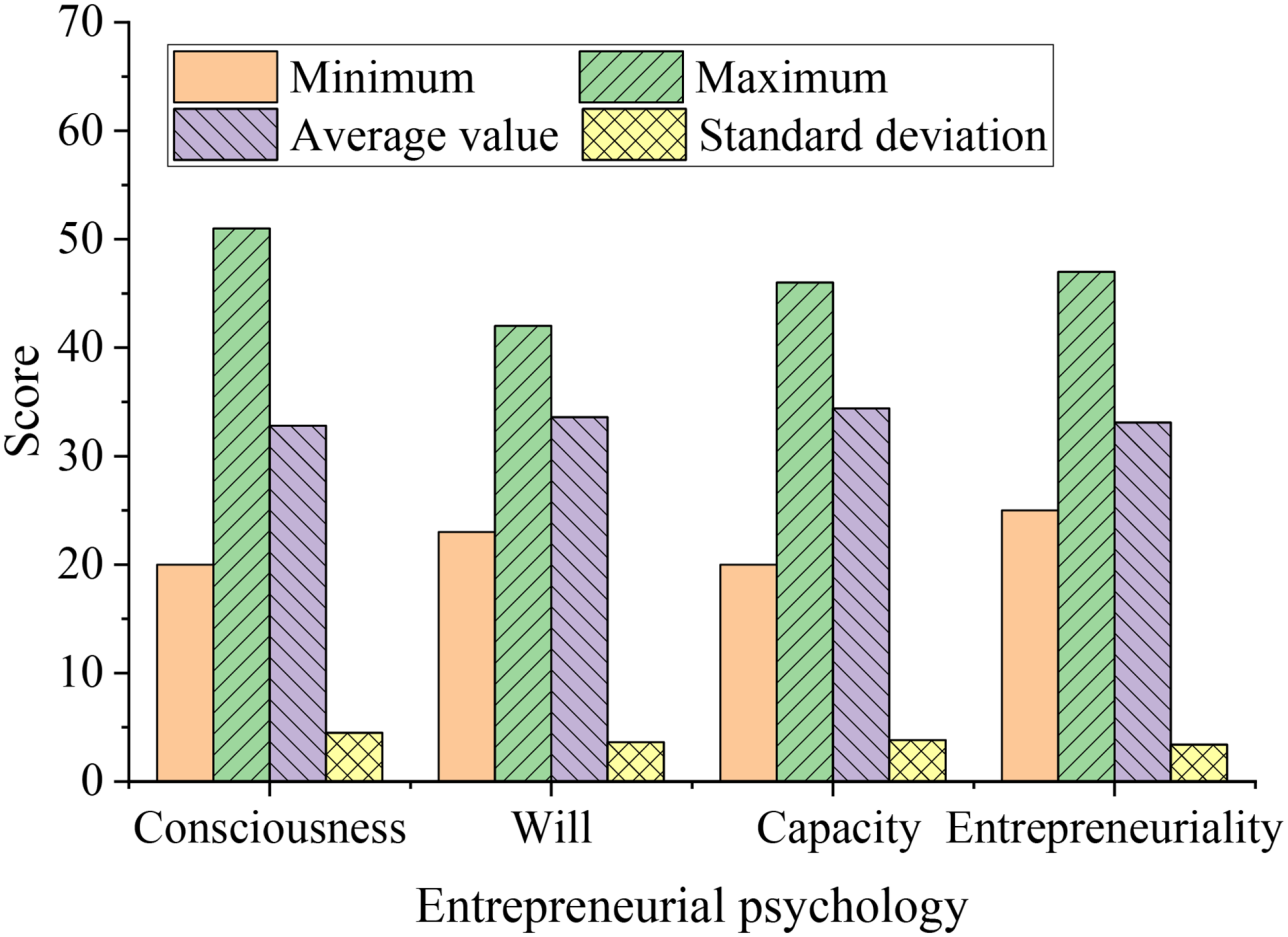
Overall situation of College Students’ entrepreneurial psychology.

After statistics of the pre-test data of the students in the experimental group and the control group, the data are analyzed by the sample *t*-test. The results are shown in Figure 2.

Figure 2 indicates that before the experiment, the highest score of each dimension of entrepreneurial psychology of the two groups of students is about 40, and the lowest score is about 20. From the perspective of a single dimension, the biggest difference between the highest score and the lowest score is entrepreneurial consciousness, indicating that entrepreneurial consciousness varies greatly due to individual differences, but the average of each dimension is between 32. Generally, the average of different dimensions is not much different, and the standard deviation is about 4.8, indicating that the difference is not very obvious.

**Figure 3:**
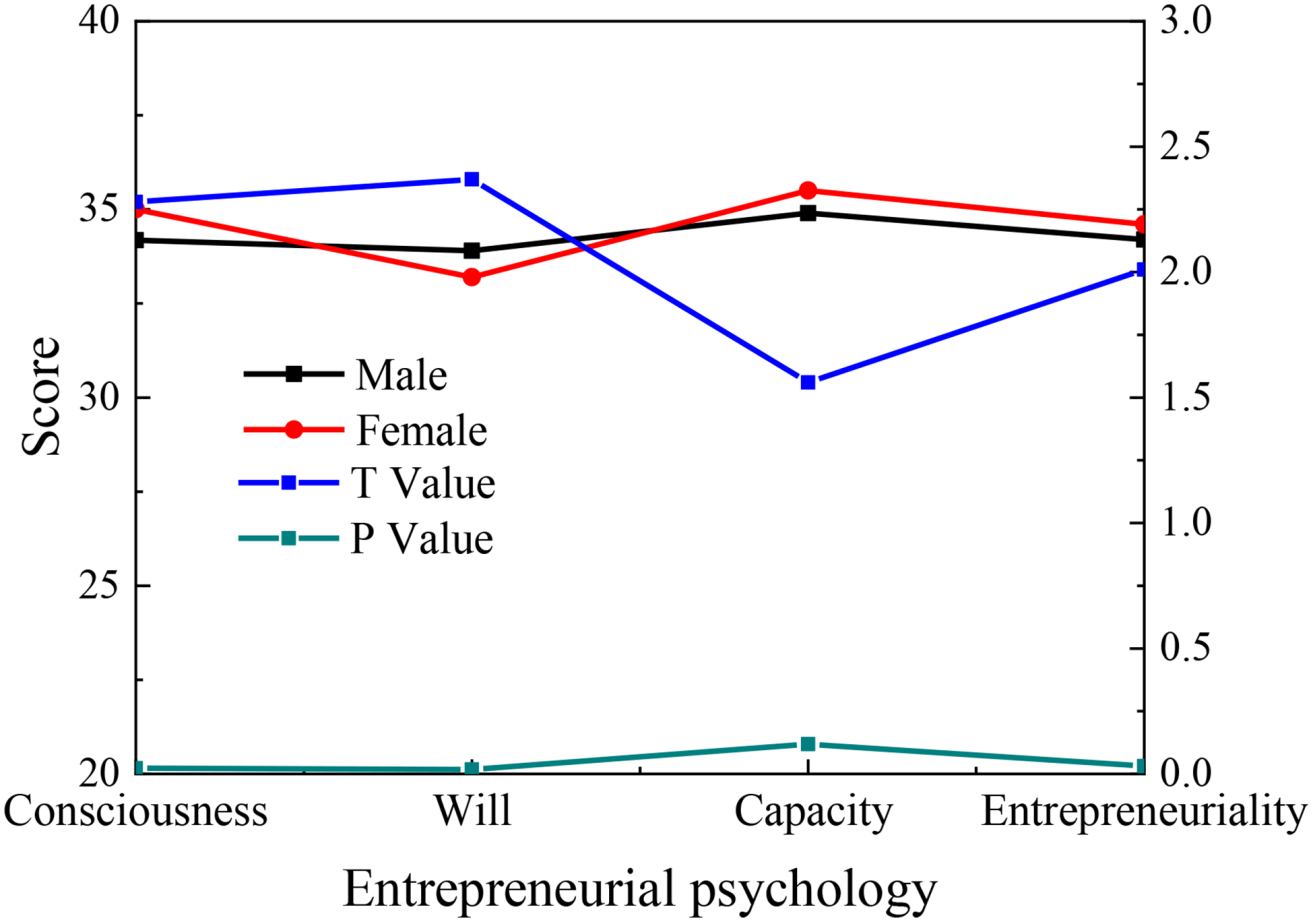
Gender differences in entrepreneurial psychology of College Students.

Independent sample *t*-test is conducted with gender as the variable, and the results are shown in Figure 3.

Figure 3 demonstrates that the gender difference of College Students is obvious in the three dimensions except for entrepreneurial capability (*p* < 0.05), which is statistically significant. In terms of entrepreneurial consciousness and entrepreneurial personality, girls score higher than boys, indicating that girls have the strong self-planning ability and distinct personalities; In terms of entrepreneurial capability, the average score of boys is higher than that of girls, indicating that boys’ personal capability and pressure resistance are higher than girls.

Overall, girls’ social status is gradually improving, and society’s recognition of women also gets higher. Women have gradually separated from the traditional role of giving birth to children and caring for the family. More women have embarked on entrepreneurship, and they are playing an increasingly important role in society. However, male College Students are more energetic and vigorous than their female peers. Their ability to accept future affairs and break through thinking conventions and solve problems is better than girls. Meanwhile, boys are more confident than girls in front of challenges. Traditionally, boys mostly live under higher pressure and competition than girls, whether as children or husbands. Boys are often given more responsibilities and obligations by society and family. Therefore, men are often asked to study hard and strive for progress. When faced with difficulties, they are more willing to take risks and constantly get twice the result with half the effort.

**Figure 4:**
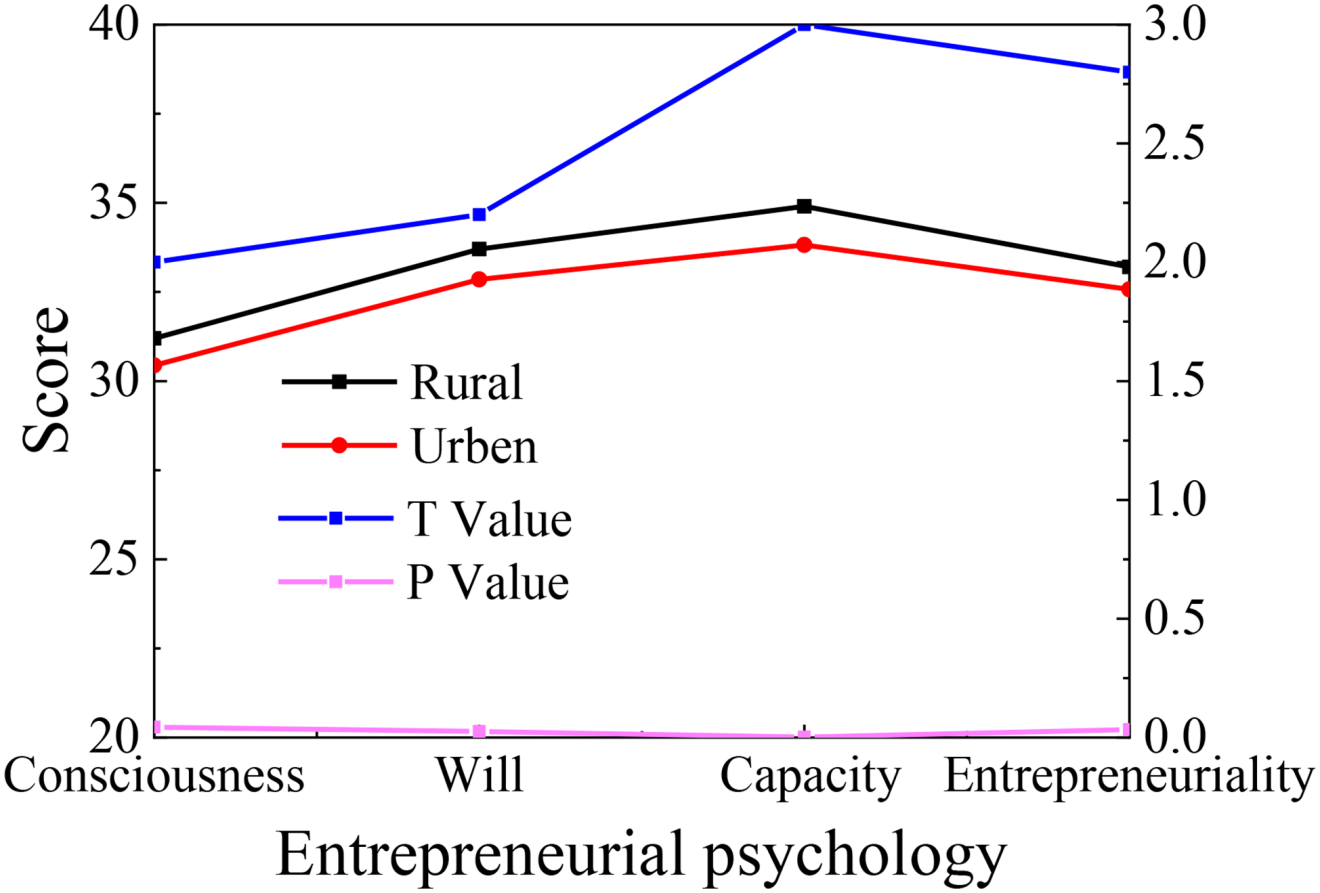
Statistics on the differences of College Students’ entrepreneurial psychology between urban and rural household registrations.

The independent sample *t*-test is conducted with urban and rural household registration as the variable, and the results are shown in Figure 4.

Figure 4 presents that there are obvious differences between urban and rural household registrations of College Students in the four dimensions of entrepreneurial consciousness, will, capability, and personality (*p* < 0.05). Specifically, the scores of students with rural household registration are higher than those with urban household registration in all dimensions, indicating that students with rural household registration are better than those with urban students in terms of entrepreneurial growth. Probably, the growth environment of students with rural household registration is relatively difficult, so they hope to break their own constraints through entrepreneurship and strive to achieve life change.

Previous literature on entrepreneurial PsyCap highlights the difference between urban and rural areas because the growth environment is an important part of an individual’s original environment. Growth environments have significantly different effects on individuals; whether it be the family background or birth area, it has a subtle influence on individual growth. Although geographical differences and environmental policies hinder most students from rural areas, they can still fully understand the entrepreneurial policies over the Internet. Thus, they have the opportunity to get in-depth contact with the entrepreneurial environment. Additionally, due to the limitations of the living environment, they are endowed with the spirit of daring to break the convention and actively seeking a breakthrough. By comparison, students from cities live a relaxed and comfortable life; they are often content with the status quo, unable to break through themselves and seek innovation.

**Figure 5:**
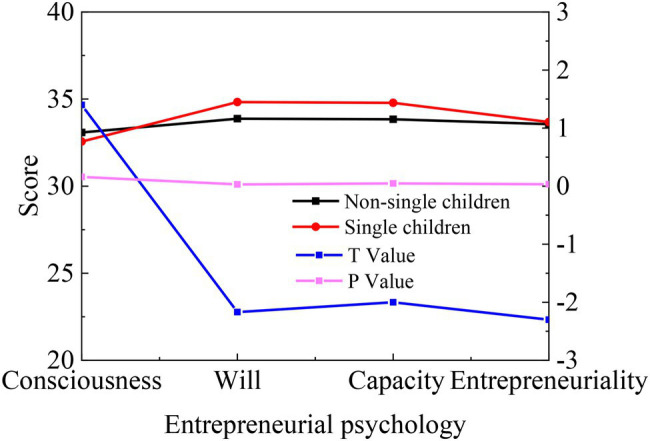
Statistics on the difference of College Students’ entrepreneurial psychology between only-child and non-only-child.

The independent sample *t*-test is conducted with the only-child-or-not as the variable, and the results are shown in Figure 5.

Figure 5 exhibits that there are significant differences between only-child and non-single child in an entrepreneurial will, entrepreneurial capability, and entrepreneurial personality (*p* < 0.05). The average score of only-child College Students is higher than that of the non-only-child. On the one hand, with the improvement of social development level and people’s material and spiritual level, the only child often enjoys higher material basis and spiritual support than non-only children. In terms of accepting and helping children, the only child’s parents often make every effort to help deal with the problems during the growth of their children. Differently, non-only child parents raise their children under a parental philosophy of “Sharing Among Family Members” and consider the overall situation. On the other hand, from the perspective of self-development, under the same conditions, the living environment and learning conditions of only children and non-only children vary dramatically. Non-only children tend to be more helpful and challenging, and they have learned to corporate and can influence each other. They often have more confidence and strength against difficulties in the entrepreneurship process, a virtuous circle of collaborative relationship development.

**Figure 6:**
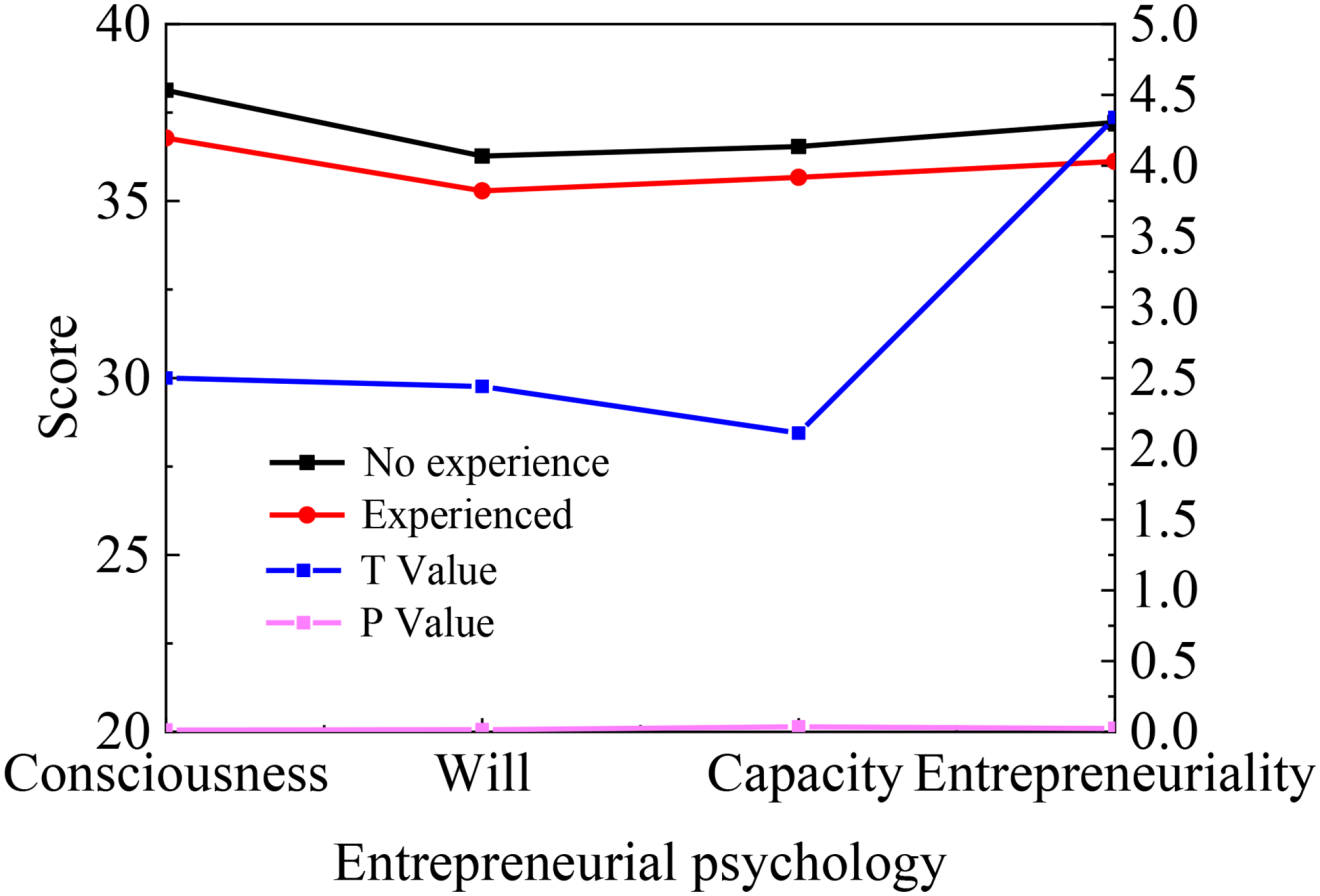
Statistics of differences of College Students’ entrepreneurial psychology in the entrepreneurial experience.

The independent sample *t*-test is conducted with entrepreneurial experience as the variable, and the results are shown in Figure 6.

Figure 6 reveals that the four dimensions of entrepreneurial consciousness, will, capability, and personality have obvious differences in terms of entrepreneurial experience (*p* < 0.05). The average score of students with entrepreneurial experience is higher than that of students without experience, indicating that entrepreneurial experience has a certain positive impact on students’ entrepreneurial growth.

Possibly, entrepreneurs in the early stage of entrepreneurship must face learning pressure, such as the shortage of resources, the obstacles of new entrants, and the dynamic changes of the company’s internal and external environment. The embarrassment of various resource shortages will force entrepreneurs to learn and obtain necessary resource information constantly. Although they continue to grow and enhance self-confidence by thinking and solving problems, the cliche behavior, decision-making, and thinking modes based on previous experiences restrict innovative entrepreneurial practices. Thereby, it also affects their ability to receive new knowledge and information.

### Post-test Results and Analysis of Inspirational Film Intervention in Entrepreneurial Psychology

**Figure 7:**
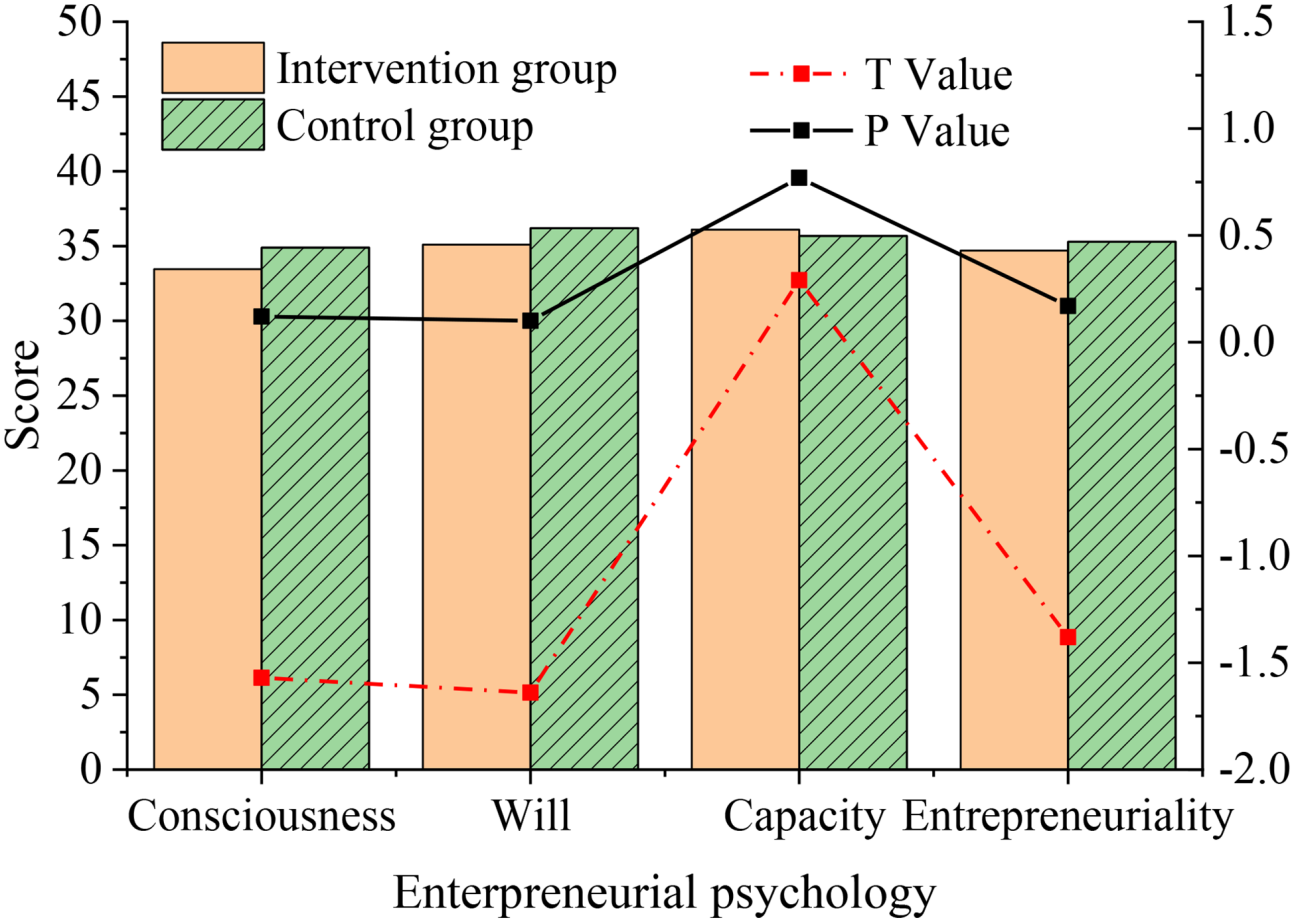
Pre-test difference of entrepreneurial psychology between intervention group and control group.

After a semester of inspirational film appreciation course, the intervention group and control group are surveyed by the QS, and the post-test results are compared with the pre-test results, as shown in Figure 7.

Figure 7 suggests that the average scores of students in the invention group and the control group are almost the same, both maintained at about 35 points, and the difference between different dimensions is not obvious (*p* > 0.05), which is not statistically significant, indicating that the entrepreneurial level of students in the two groups is basically at the same level, ensuring the fairness of the experiment.

**Figure 8:**
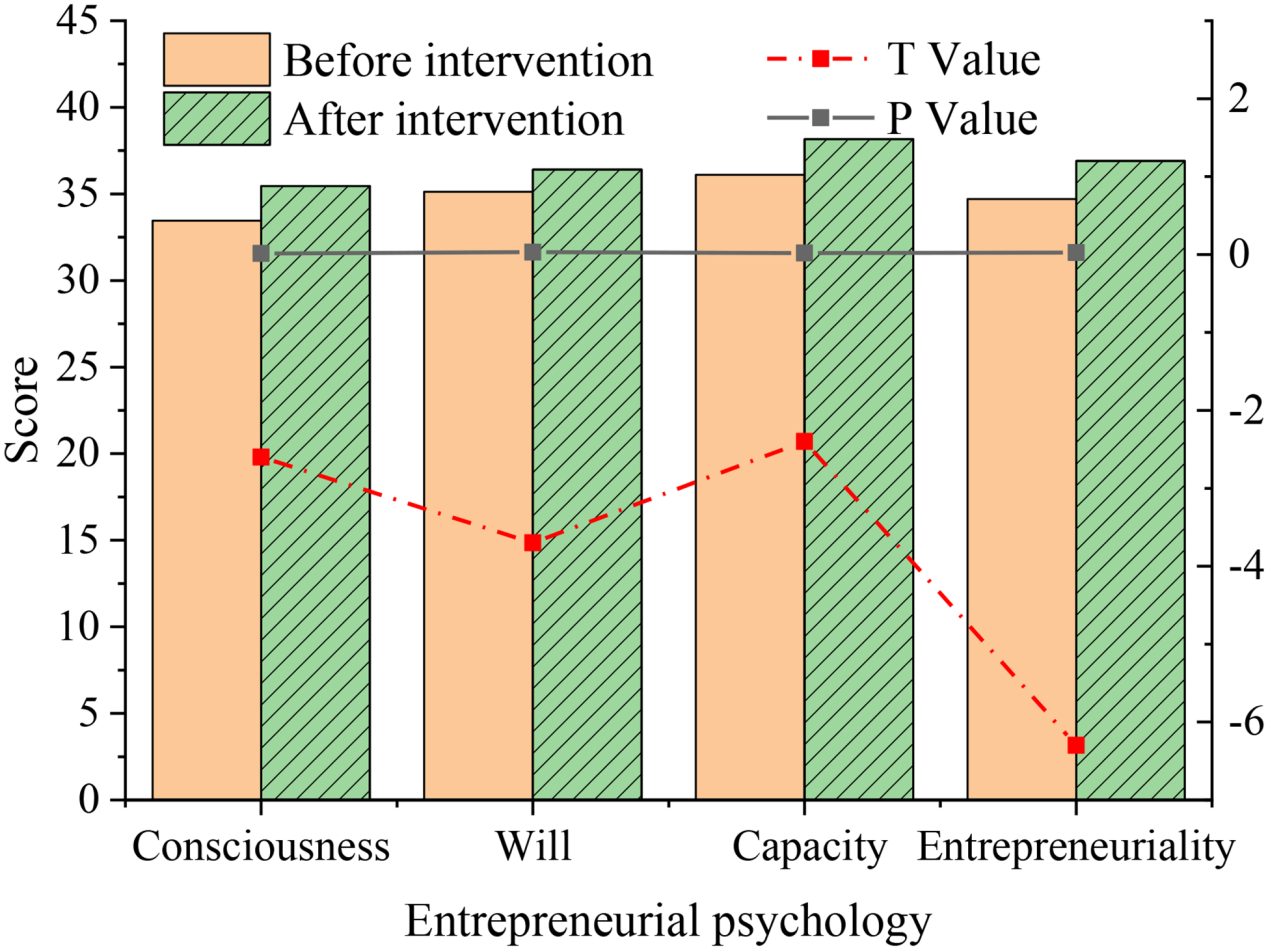
Statistics on the difference of entrepreneurial level after film appreciation in the intervention group.

The comparison of scores of students in the intervention group before and after the intervention is shown in Figure 8.

Figure 8 implies that after the intervention of inspirational film, the scores of all dimensions of the entrepreneurial level of students in the intervention group have been slightly improved compared with the situation before the intervention, and the differences in the four dimensions are obvious (*p* < 0.05), which is statistically significant, indicating that the intervention of inspirational film has a certain positive impact on students’ entrepreneurial level.

**Figure 9:**
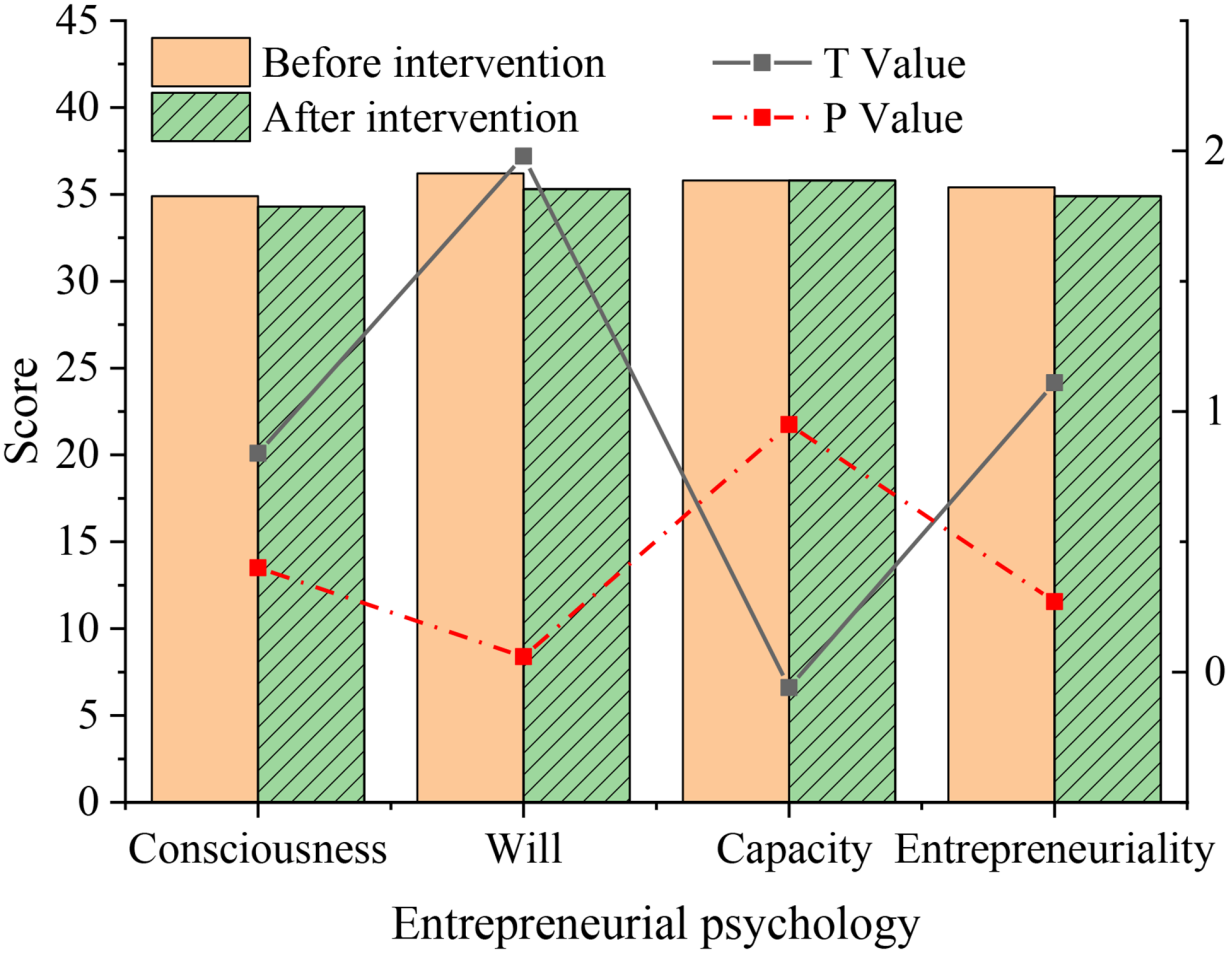
*T*-test results of paired samples in each dimension of pre-test and post-test in the control group.

Additionally, the comparison of pre-test and post-test results of the control group is shown in Figure 9.

Figure 9 shows the test results of students in the control group before and after the intervention; the scores of all dimensions of entrepreneurial level have not changed very much, only fluctuated slightly, and these differences are not very obvious (*p* > 0.05), indicating that there is no significant change in the entrepreneurial level of students in the control group before and after the experiment.

**Figure 10:**
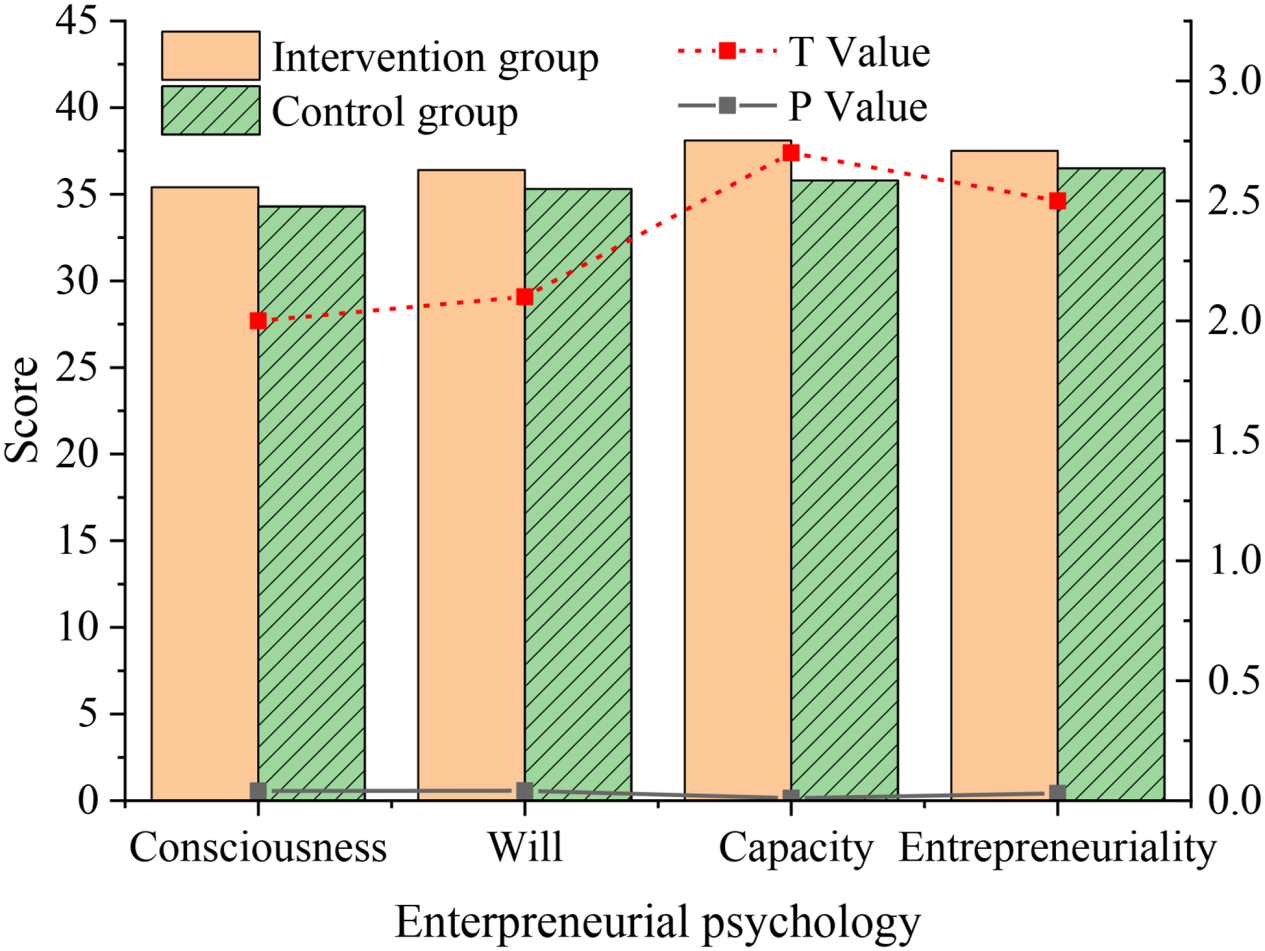
Differences in the post-test level of entrepreneurial psychology between the intervention group and the control group.

The comparison of the overall situation of the students in the intervention group and the control group after the experiment is shown in Figure 10.

Figure 10 demonstrates that the average scores of students in the intervention group in entrepreneurial awareness, entrepreneurial will, entrepreneurial capability, and entrepreneurial personality are 35.45, 36.41, 38.17, and 37.22, respectively, which are higher than those in the control group, and the difference is obvious (*p* < 0.05), indicating that the students in the intervention group have greatly improved in all dimensions of entrepreneurial level compared with the control group, The overall entrepreneurial level is better than the control group. The intervention of inspirational films has a positive impact on improving students’ entrepreneurial level. This also proves that the inspirational film has a significant impact on the entrepreneurial PsyCap of college students. It is necessary and feasible to supplement the content of inspirational film appreciation in the PsyH education curriculum. The two assumptions proposed are tenable.

## Conclusion

Under the background of multiculturalism, an innovative investigation and research are conducted on College Students’ entrepreneurial psychology based on positive psychology theory. The positive impact of inspirational films and roles is explored on College Students’ entrepreneurial PsyCap. Here, a qualitative analysis is conducted on local College Students through QS, intervention method, and independent-sample *t*-test. The survey results show that: (1) The gender differences of college students are statistically significant in the three dimensions except for entrepreneurial ability. Girls score higher than boys in terms of entrepreneurial consciousness and entrepreneurial personality. There are obvious differences in the four dimensions of entrepreneurial consciousness, will, ability, and personality among College Students in urban and rural areas. Thus, the specific scores imply that the scores of students with rural accounts are higher than those of urban accounts in all dimensions; whether they have certain entrepreneurial experience has obvious differences in four dimensions: consciousness, will, ability, and personality. The average score of students with entrepreneurial experience is higher than green hands. (2) The intervention of inspirational films has a certain positive impact on students’ entrepreneurial level.

Shortcomings of this paper: in terms of College Students’ entrepreneurial factors, although all sample students are controlled with the same major, some factors, such as age and family economic situation, are involved, which needs to be improved in the follow-up survey. With the advent of the entrepreneurial era, more College Students are joining the entrepreneurial team. The IEE in higher institutions still needs to continue to improve the teaching effect and scientifically utilize excellent inspirational films to guide students to improve their entrepreneurial level, thereby expanding its social influence.

## Data Availability Statement

The raw data supporting the conclusions of this article will be made available by the authors, without undue reservation.

## Ethics Statement

The studies involving human participants were reviewed and approved by Hangzhou Dianzi University Ethics Committee. The patients/participants provided their written informed consent to participate in this study. Written informed consent was obtained from the individual(s) for the publication of any potentially identifiable images or data included in this article.

## Author Contributions

All authors listed have made a substantial, direct, and intellectual contribution to the work and approved it for publication.

## Funding

This research is funded by Humanity and Social Science Youth foundation of Ministry of Education of China, 21YJC760102. This work was supported by National Ethnic Affairs Commission of the People’s Republic of China Project Political Security Governance in Minority Areas from the Perspective of Overall Security (No. 2021-GMB-009).

## Conflict of Interest

The authors declare that the research was conducted in the absence of any commercial or financial relationships that could be construed as a potential conflict of interest.

## Publisher’s Note

All claims expressed in this article are solely those of the authors and do not necessarily represent those of their affiliated organizations, or those of the publisher, the editors and the reviewers. Any product that may be evaluated in this article, or claim that may be made by its manufacturer, is not guaranteed or endorsed by the publisher.
